# Those Virtual People all Look the Same to me: Computer-Rendered Faces Elicit a Higher False Alarm Rate Than Real Human Faces in a Recognition Memory Task

**DOI:** 10.3389/fpsyg.2018.01362

**Published:** 2018-08-03

**Authors:** Jari Kätsyri

**Affiliations:** Brain and Emotion Laboratory, Department of Cognitive Neuroscience, Maastricht University, Maastricht, Netherlands

**Keywords:** artificial faces, face recognition, face memory, face inversion, uncanny valley hypothesis

## Abstract

Virtual as compared with real human characters can elicit a sense of uneasiness in human observers, characterized by lack of familiarity and even feelings of eeriness (the “uncanny valley” hypothesis). Here we test the possibility that this alleged lack of familiarity is literal in the sense that people have lesser perceptual expertise in processing virtual as compared with real human faces. Sixty-four participants took part in a recognition memory study in which they first learned a set of faces and were then asked to recognize them in a testing session. We used real and virtual (computer-rendered) versions of the same faces, presented in either upright or inverted orientation. Real and virtual faces were matched for low-level visual features such as global luminosity and spatial frequency contents. Our results demonstrated a higher response bias toward responding “seen before” for virtual as compared with real faces, which was further explained by a higher false alarm rate for the former. This finding resembles a similar effect for recognizing human faces from other than one's own ethnic groups (the “other race effect”). Virtual faces received clearly higher subjective eeriness ratings than real faces. Our results did not provide evidence of poorer overall recognition memory or lesser inversion effect for virtual faces, however. The higher false alarm rate finding supports the notion that lesser perceptual expertise may contribute to the lack of subjective familiarity with virtual faces. We discuss alternative interpretations and provide suggestions for future research.

## Introduction

Virtual environments and augmented realities are not only changing the way we perceive “reality” but also the way we perceive and interact with its real and virtual inhabitants. Even though many individuals frequently encounter realistic virtual characters in video games and other media (e.g., animation films), most of our perceptual expertise is arguably still shaped by our interactions with our biological companions. For example, parents' faces are among the very first things newborns encounter after being born, and an innate interest in human faces remains characteristic to typically developing children. According to the “uncanny valley” hypothesis (Mori, 1970), artificial entities bearing a near-identical resemblance to real humans elicit a sense of uneasiness, characterized by lack of familiarity and even feelings of eeriness, even though increasingly realistic artificial characters in general tend to elicit more positive responses. Although empirical evidence for the pronounced unfamiliarity of near-human entities still appears inconsistent, the bulk of studies support the overall positive association between realism and familiarity (Kätsyri et al., 2015). Abundant exposure to real human faces from an early life could possibly explain the lack of subjective familiarity with virtual faces. In this case, human observers should conversely possess lesser perceptual expertise in processing virtual as compared with real faces. In the present study, we investigate whether participants are indeed impoverished in processing virtual faces—that is, faces that are close yet distinguishable computer-generated approximations of real faces.

Several lines of evidence suggest that the human visual system possesses perceptual expertise with faces that is shaped by exposure (even though social-cognitive motivational factors may also play a role; e.g., Bernstein et al., 2007). One of the earliest and best documented examples is the tendency for perceivers to have more accurate recognition memory for faces from one's own ethnic group in comparison to faces from other ethnicities (Meissner and Brigham, 2001; Young et al., 2012). Algorithmic analysis of three-dimensional head scans has provided support for one prerequisite of this effect, the existence of ethnicity-characteristic facial features (O'Toole et al., 1991; Salah et al., 2008). More accurate recognition of own-ethnicity faces has become widely known as the other-race effect, own-race bias, or cross-race effect. Such terms may be misleading, however, given that this effect is not only biologically determined. For example, individuals from one country who were adopted into another country at an early age showed a reversal of the effect such that they recognized faces originating from their adoption country better than faces originating from their birth country (Sangrigoli et al., 2005). In a similar vein, training has been shown to reduce the recognition disadvantage for other-ethnicity faces (e.g., Hills and Lewis, 2006; Tanaka and Pierce, 2009). Guiding participant's attention to features that are characteristic of other-ethnicity faces can also eliminate the effect (Hills et al., 2013). Such findings both exemplify the malleability of the other-ethnicity effect and argue against its biologically or racially determined origins. Furthermore, the term “race” itself has been called into question both in biology and neuroscience because of its inexact and prejudiced nature (Yudell et al., 2016; Cubelli and Della Sala, 2017). Hence, following Valentine et al. (2016), we refer to this phenomenon as the own-ethnicity bias (OEB). Biases resembling the OEB have been demonstrated also for other variables besides ethnicity—for example, men have better recognition memory for male than female faces, whereas the opposite holds true for women (Wright and Sladden, 2003). This suggests that also the processing of own-gender faces may be fine-tuned by possibly greater exposure to same-gender individuals.

Most studies documenting the OEB effect have used a standard old-new recognition memory paradigm in which participants are first asked to memorize a set of faces and then tested for their ability to discriminate between previously seen (target) and previously unseen (distractor) faces (Meissner and Brigham, 2001). Typical findings show a “mirror effect” in which own-ethnicity faces yield a higher proportion of hits (targets identified as previously seen) and a lower proportion of false alarms (distractors identified as previously seen) as compared to other-ethnicity faces (e.g., Meissner et al., 2005). Inflated false alarm rate for other-ethnicity faces means that people tend to confuse individuals from other ethnic groups readily with one another—a phenomenon which could be characterized anecdotally with the statement “They all look the same to me” (e.g., Ackerman et al., 2006).

The finding that ethnicity modulates not only the proportion of hit rates but the proportion of false alarms as well has been previously explained in the framework of the face-space coding model of Valentine (Valentine, 1991; Valentine et al., 2016). Generally speaking, this model suggests that faces are represented mentally in a multidimensional space. These dimensions can correspond to any features that serve to discriminate between individuals (e.g., mouth shape or inter-ocular distance); however, they are not explicitly defined by the model. Face-space model posits that these dimensions are selected and scaled to optimize discrimination of frequently encountered faces. Hence, these dimensions are optimized for own-ethnicity faces that are by definition encountered frequently but, assuming infrequent encounters with other ethnic groups, they are less efficient for encoding differences between other-ethnicity faces (cf. Valentine, 1991). As a result, different other-ethnicity faces can share identical values on several dimensions, which means that they end up being clustered more densely in the face-space than own-ethnicity faces. Conversely, encountering an other-ethnicity face activates more exemplars in the face-space, which makes it more difficult to determine whether that face was in fact encountered previously or whether it is merely similar to other previously seen faces. According to the model, this ultimately generates a higher proportion of false alarms for other-ethnicity faces as compared with own-ethnicity faces.

Inversion effect, or the slower and much less accurate recognition of upside-down as compared with upright faces, is considered one of the hallmarks of perceptual expertise with faces or other well-learned objects (Maurer et al., 2002). Allegedly, inversion has a greater effect on configural (or holistic; perceiving relations among features) than featural (or piece-meal; processing individual features) processing of faces. A possible alternative explanation based on the face-space model could be that face inversion, similarly as many other impairments (e.g., blurring, adding noise, or presenting photographic negatives), simply introduces noise to face encoding (Valentine, 1991). Although the interaction between the OEB and inversion is not entirely uncontroversial (for a review, see Young et al., 2012), evidence exists for a greater inversion effect in own-ethnicity than other-ethnicity faces (e.g., Rhodes et al., 1989; Vizioli et al., 2011). Such findings are consistent with the notion that individuals possess more perceptual expertise with own- as compared with other-ethnicity faces. Furthermore, they contradict the notion that inversion would simply add noise to face encoding because if this were the case, inversion should elicit even greater impairment on the already impoverished encoding of other-ethnicity faces. Hence, these findings also suggest that other-ethnicity faces may be processed in a more featural or piece-meal fashion than own-ethnicity faces.

We next turn to the question of whether the processing of virtual faces could be similar to other-ethnicity faces when it comes to face encoding; or more specifically, mirror and inversion effects in face recognition. First, however, we note that contemporary computer-rendering methods do not yet tap face processing expertise fully to the same extent than real human faces. Arguably, FaceGen Modeler (Singular Inversions) is one of the most versatile and most commonly used programs for face perception experiments (e.g., Cook et al., 2012; MacDorman et al., 2013; Balas and Pacella, 2015; Crookes et al., 2015). This program can be used to create both reconstructions of real faces and randomly generated novel faces in a parametric space derived from a large number of three-dimensional face scans (Blanz and Vetter, 1999). Recently, Crookes et al. (2015) contrasted the OEB for real and FaceGen-generated virtual faces using face recognition memory and perceptual discrimination tasks. Their results demonstrated reduced accuracy for virtual faces in both tasks, and an attenuated OEB for virtual as compared with real faces in the recognition memory task. These findings hence show that virtual faces based on FaceGen software are close but not perfect reconstructions of real human faces, and that they elicit a similar but weaker OEB effect than real faces. In a similar recent study, Balas and Pacella (2015) contrasted recognition memory and discrimination accuracy between virtual and real faces, where the former were again generated by FaceGen. Their results demonstrated that participants were less accurate in recognizing virtual faces in comparison to real faces. Similarly, participants were less accurate in matching two faces to an immediately preceding face image in an ABX matching task.

Even though these two studies demonstrate that FaceGen-generated virtual face stimuli perform less efficiently than real human faces, it is questionable whether their results can be generalized to other virtual faces as well. An important distinction between other-ethnicity faces and virtual faces is that whereas other-ethnicity faces may possess genuine ethnicity-characteristic features (cf. O'Toole et al., 1991; Salah et al., 2008), virtual faces are recognized as “virtual” only when they fail to replicate some characteristics of their reference stimuli (real faces). For example, it is possible that FaceGen-generated virtual faces are artifactual or less detailed replications of real faces, or that they differ from real faces in terms of brightness, contrast, or colors. The extent to which such trivial low-level differences could explain previously observed differences between real and virtual faces is presently not known.

An unfortunate characteristic of all virtual faces is that they can in fact have very little in common. This raises the question of whether it is at all justifiable to consider virtual faces as a unified category of research stimuli. Previous studies investigating continua from virtual to real faces have, however, shown that virtual faces are perceived categorically; that is, equally spaced image pairs are discriminated better when they straddle the virtual–real category boundary than when they reside on the same side of it (Looser and Wheatley, 2010; Cheetham et al., 2011). Changes in virtual–real category in sequentially presented faces are also known to elicit fMRI responses in category learning and uncertainty related neural networks (Cheetham et al., 2011). These findings suggest that virtual and real faces are typically perceived as distinct categories, similarly as faces of different species (Campbell et al., 1997) or faces of different ethnic groups (Levin and Angelone, 2002). Furthermore, exposure may also modulate categorization and evaluation of virtual faces. Burleigh and Schoenherr (2015) demonstrated that more frequent exposure to specific morph levels between two computer-generated faces improves categorization accuracy for these levels. Frequency-based exposure was also found to modulate participants' subjective ratings, albeit at a statistically non-significant level.

In the present investigation, we operationalize virtual faces using FaceGen but also correct them for most obvious artifacts, and match real and virtual faces with respect to specific low-level visual features. The purpose of this procedure is to increase the generalizability of present results beyond that of a specific computer-rendering method. A justifiable concern after such matching procedure, however, is whether real and virtual faces can still be discriminated from each other. Trivially, if computer-generated images were sufficiently similar to real images, the two would be indistinguishable from each other even by experts (cf. Lehmuskallio et al., 2018).

## Study 1

In this study, we first investigate whether real and virtual face images can be differentiated from each other even after they have been matched for the following low-level visual features: spatial frequency contents (level of details), brightness, contrast, and colors. Most obvious artifacts are also removed from the virtual faces. Importantly, this matching is done for whole images, that is, at global level. It is possible that even after such global-level matching, local features such as the shapes of individual features may serve to differentiate between real and virtual faces. Subtle artifacts may also remain in the local features of virtual faces. Furthermore, it is possible that low-level visual features still vary at the local level after they have been matched globally. For example, it is possible that nose and eye region brightness might differ in two images even though their averages remained the same. Conversely, we predict that real and virtual faces can still be differentiated from each other based on any of such local differences. Hence, we make the following hypothesis for Study 1:

H1: Real and virtual faces can be differentiated from each other, even after global-level matching for spatial frequency contents, brightness, contrast, and colors.

In practical terms, colors add extra complications to psychophysical experiments given that one has to consider matching three color channels between images instead of only one luminosity channel. Hence, our secondary research question is whether colors truly contribute to differentiating virtual from real faces. Previous studies suggest that real and virtual faces are easier to discriminate from color than grayscale images (Fan et al., 2012, 2014; Farid and Bravo, 2012). However, given that these studies used different image sets for real and virtual faces, it is conceivable that these results would reflect differences between the employed image samples. Hence, we also aim to test the following secondary hypothesis.

H2: Real and virtual faces are discriminated better from color than grayscale images.

### Methods

#### Participants

Participants were 48 (29 women) university students whose age ranged from 18 to 30 years (*M* = 20.9 years). All participants identified themselves as Caucasian in ethnic origin. Participants signed to the study anonymously using the SONA system (http://www.sona-systems.com) of Maastricht University, and received course credit in compensation for their participation. All participants gave written informed consent in accordance with the Declaration of Helsinki. The present studies were reviewed and approved by the ethics committee of the Faculty of Psychology and Neuroscience.

#### Design

The study had a 2 (face type: real, virtual) × 2 (spatial frequency matching: strict, lenient) × 2 (colors: grayscale, color) within-subjects design.

#### Stimuli

**Figure 1:**
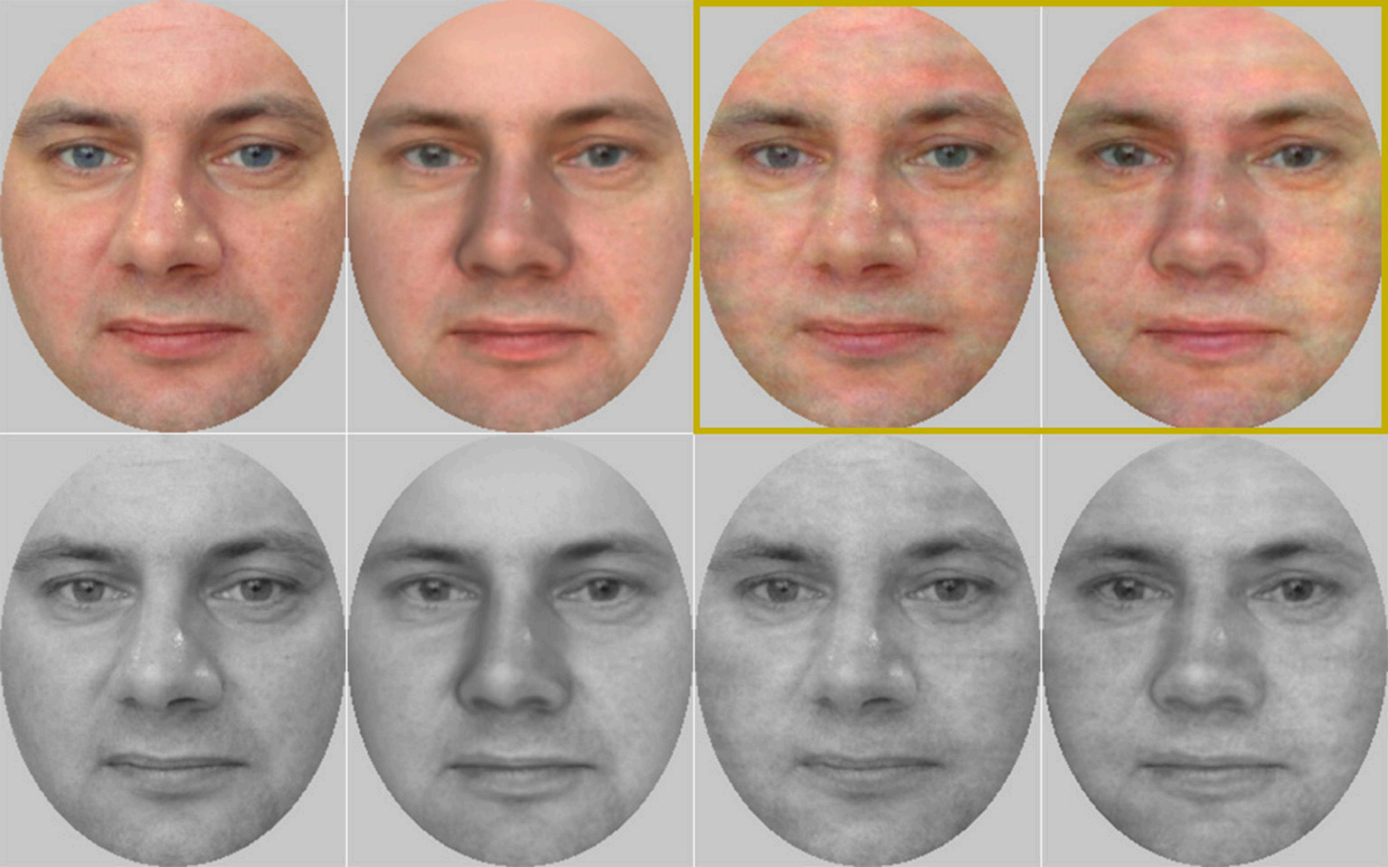
Sample images for one actor extracted from the Glasgow face set (Burton et al., 2010). Upper row: color images, bottom row: grayscale images. From left to right: real face after lenient spatial frequency matching, virtual face after lenient matching, real face after strict matching, and virtual face after strict matching. Only strictly matched color images (surrounded by dark-yellow square on the top-right corner) were used in Study 2.

Research stimulus samples are shown in Figure 1. Real face stimuli were 12 neutral face images (half female) from Glasgow (Burton et al., 2010) and Radboud (Langner et al., 2010) face image sets. Virtual face stimuli were created using FaceGen Modeler (Singular Inversions; Version 3.13). Real faces (frontal images only) were imported into FaceGen, and an initial alignment was provided using a number of feature points. Reconstructed and original faces were aligned and matched with each other to the extent possible with respect to small variations in head position, gaze direction, and facial expression. Major artifacts (in particular, black line between the lips) were corrected in Photoshop (Adobe; Version CS6). All images were oval-masked to conceal external features (ears and hair), which would otherwise have been clearly unrealistic in the virtual stimuli. Final images were 246 × 326 pixels in size.

All further image manipulations were carried out in Matlab (The Mathworks Inc.; Version R2016a). Grayscale images were produced by weighting original RGB channel values. Inhouse functions based on SHINE toolbox (Willenbockel et al., 2010) were used for standardizing images. Two methods were used for matching energy at different spatial frequencies across the images: matching the whole Fourier spectra (“strict matching”) and matching only the rotational average of the Fourier spectra (“lenient matching”)—for details, please refer to Willenbockel et al. (2010). We used the latter matching procedure in place of original (non-matched) images, given that leniently matched and original images were practically identical and led to similar results in pilot tests. Prior to spatial frequency matching, image backgrounds were substituted by the average pixel intensity values within the masked face regions to reduce sharp transitions in the images. Mean and standard deviations for the pixel values within the masked region were standardized across images, and backgrounds in the final images were substituted with a constant gray color. For color images, image matching was carried out separately for each RGB channel (cf. Kobayashi et al., 2012; Railo et al., 2016).

#### Procedure

This study was carried out as an online evaluation, which was programmed and hosted through Qualtrics platform (http://www.qualtrics.com). Only participants using a laptop or a desktop computer with a sufficiently large display (minimum 12”) were included. A total of 96 stimuli (8 conditions × 12 actors) were presented in a pseudo-randomized order. Participants were asked to identify whether each stimulus portrayed a human or a virtual face in a one-interval forced choice task with two response alternatives. Participants were also asked to indicate how confident they were of their choice using a 5-step Likert scale (1—uncertain, 2—somewhat uncertain, 3—somewhat certain, 4—certain, 5—absolutely certain). The questionnaire was self-paced, but participants were instructed to answer each question as quickly and as accurately as possible. Participants were required to carry out the questionnaire in a single session without breaks.

#### Preprocessing

Hit and false alarm rates for the identification task were transformed into sensitivity index *d'* and response bias index *c*, calculated according to signal detection theory using the following standard formulae (Stanislaw and Todorov, 1999; Chapter 2 in Stevens and Pashler, 2002).

$$\begin{array}{lll} d^{\prime } & = & z\left(H\right)-z\left(F\right)\\ c\; & = & \;-\frac{1}{2}\left(z\left(H\right)+z\left(F\right)\right) \end{array}$$

Here, hit rate (H) refers to the proportion of real faces identified correctly as human, and false alarm rate (F) refers to the proportion of virtual faces identified incorrectly as human. Following the guidelines of Stanislaw and Todorov (1999), H and F were corrected using log-linear method to avoid incalculable values. In the present study, *d*′ reflects the extent to which participants were able to differentiate between real and virtual faces. Theoretically, *c* can be understood as the difference between participants' response criterion and neutral point where neither response alternative is favored. In the present one-interval task, response criterion can be interpreted in terms of “human” responses. Positive values refer to more conservative response criterion or a tendency to respond “virtual,” whereas negative values refer to more liberal response criterion or tendency toward responding “human” for all faces.

### Results and discussion

**Figure 2:**
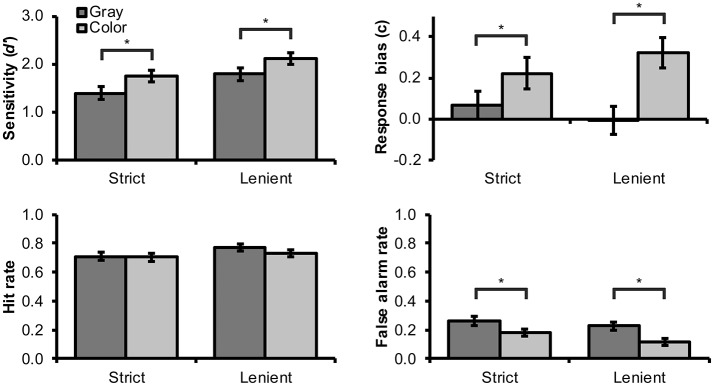
Mean (and SEM) values for *d'* sensitivity index, c response bias index, hit rate, and false alarm rate for the identification task by color and spatial frequency matching conditions. Statistically significant differences between color and grayscale images are denoted with an asterisk (“*”).

Results for different conditions are illustrated in Figure 2. For testing H1, we first compared *d*′ scores to zero using one-sample *T*-tests. Test results showed that *d*′ scores were significantly above zero in all conditions, *T*_(47)_ > 10.01, *p* < 0.001, Cohen's *d* > 1.44, which indicates that real and virtual faces were clearly differentiated from each other in all experimental conditions. Next, a 2 × 2 within-subjects ANOVA was used to assess the influence of color and spatial frequency matching on *d*′ scores. Significant main effects were observed for spatial frequency matching, *F*_(1, 47)_ = 26.08, *p* < 0.001, *n*_*p*_^2^ = 0.36, and color, *F*_(1, 47)_ = 25.34, *p* < 0.001, *n*_*p*_^2^ = 0.35. Strict matching elicited lower *d*′ sensitivity scores than lenient matching (Figure 2). As predicted by H2, color images elicited higher *d*′ scores than grayscale images.

For completeness, we also analyzed response bias values using similar analysis. We observed a significant main effect for color, *F*_(1, 47)_ = 25.17, *p* < 0.001, *n*_*p*_^2^ = 0.35, and a significant interaction between spatial frequency matching and color, *F*_(1, 47)_ = 5.25, *p* = 0.027, *n*_*p*_^2^ = 0.10. Specifically, color images elicited higher *c* values (bias toward responding “virtual”) than grayscale images. This effect was weaker for strictly than leniently filtered images (Figure 2), which may suggest that it was partly obscured by the strict filtering procedure (however, similar effect was not observed for false alarm rates; see below). To understand these results better, we next analyzed hit and false alarm rates individually. Color images elicited a lower proportion of false alarms than grayscale images, *F*_(1, 47)_ = 30.38, *p* < 0.001, *n*_*p*_^2^ = 0.39. In other words, when shown in color, virtual faces were mistaken less frequently for real faces. There was also a non-significant tendency toward a higher false alarm rate for strictly rather than leniently filtered faces, *F*_(1, 47)_ = 3.91, *p* = 0.054, *n*_*p*_^2^ = 0.08. Interaction between spatial frequency matching and color was not significant, *F*_(1, 47)_ < 1, *p* = 0.432, *n*_*p*_^2^ = 0.01. No significant effects were observed for hit rates, which suggests that both sensitivity and response bias findings were driven mainly by changes in false alarm rates. We interpret these results to mean that colors are particularly important for recognizing virtual faces as artificial but have a smaller role for the correct recognition of real faces as human.

Confidence ratings were additionally analyzed using a 2 (color) × 2 (spatial frequency matching) × 2 (face type) within-subjects ANOVA. The results showed a significant main effect for spatial frequency matching, *F*_(1, 47)_ = 75.30, *p* < 0.001, η_*p*_^2^ = 0.62, and a significant interaction effect for color and face type, *F*_(1, 47)_ = 7.19, *p* = 0.010, *n*_*p*_^2^ = 0.13. Strict as compared with lenient spatial frequency matching elicited generally lower confidence ratings regardless of face type (*M* = 3.47 and 3.77, *SD* = 0.48 and 0.49). Simple effect tests showed that confidence ratings for color and grayscale images differed only for virtual faces (*p* = 0.003). Specifically, participants rated higher confidence when categorizing virtual faces from color rather than grayscale images (*M* = 3.81 and 3.56, *SD* = 0.49 and 0.55). This finding further corroborates the importance of colors for recognizing virtual faces.

Not surprisingly, the present results showed that the “strict” spatial frequency matching procedure elicited lower discrimination performance and lower confidence ratings than the “lenient” procedure, which we considered analogous to unmatched stimuli. At the same time, our results confirmed that highly realistic virtual faces (cf. Figure 1) could still be differentiated from real human faces relatively easily even after the strict matching procedure. Given that any experimental comparison between unmatched real and virtual faces would be confounded by differences in spatial frequency contents (e.g., overall lack of details in virtual faces), we hence decided to adopt the strict matching procedure for our second experiment. Our other findings replicate the previous finding (Fan et al., 2012, 2014; Farid and Bravo, 2012), with slightly better controlled stimuli, that real and virtual faces are differentiated better and with higher confidence from color as compared with grayscale images. A closer inspection of false alarm rates as well as participants' confidence ratings suggested that colors are particularly important for the correct recognition of virtual faces. Interestingly, visual inspection of Figure 2 would suggest that color has a roughly similar effect on discrimination accuracy than the present choice of spatial frequency matching. Hence, we conclude that adopting color rather than grayscale images can be used to compensate for the loss of discrimination accuracy caused by strict spatial frequency matching.

## Study 2

In the second study, we continue to investigate whether our rigorously matched virtual faces tap perceptual expertise similarly as real human faces. We expect to observe a similar mirror pattern as in previous OEB studies in which other-ethnicity faces elicited both a lower proportion of hits and a higher proportion of false alarms than own-ethnicity faces (e.g., Meissner et al., 2005). Following this pattern, aggregate measures based on hits and false alarms have previously indicated lower discrimination accuracy (discrimination between previously seen and novel faces) and lower response bias (overall tendency to respond “previously seen” to all faces) for other-ethnicity faces. We predict analogous effects for virtual faces. That is,

H1: Virtual faces will elicit lower discrimination accuracy than real faces.

H2: Virtual faces will elicit lower response bias than real faces.

Previous findings suggest that inflated false alarm rate for other-ethnicity faces—or the “They all look the same to me” phenomenon—is a major factor driving the OEB effect. One explanation for this is that facial encoding dimensions in the face-space model of Valentine (Valentine, 1991; Valentine et al., 2016) are optimized for discriminating frequently seen own-ethnicity faces but that they are suboptimal when it comes to the discrimination of other-ethnicity faces. Assuming that virtual faces contain sufficiently different or distorted features with respect to real human faces, we predict a similar effect for virtual faces as well. That is, we predict that:

H3: Virtual faces will elicit a higher proportion of false alarms than real faces.

In the present study, we also investigate the effect of inversion on the recognition of virtual faces. In their previous study, Balas and Pacella (2015) observed an equally large inversion effect for virtual and real faces in a perceptual discrimination task. Performance was close to ceiling level for both upright and inverted faces, however, which leaves open the possibility that a more difficult task might be more sensitive to differential inversion effects in real and virtual faces. A diminished inversion effect for virtual faces could be taken as evidence that virtual faces are processed in a more piece-meal and less “face-like” manner than real faces. Here we test the following prediction:

H4: Real faces will elicit a greater inversion effect as measured with discrimination accuracy than virtual faces.

Previous factor-analytic research on participants' self-reports have demonstrated that the typicality (or distinctiveness) of faces is composed of two orthogonal components: memorability and general or context-free familiarity (Vokey and Read, 1992; Meissner et al., 2005). For the present context, it is interesting that the latter factor combines familiarity with attractiveness and likability. That is, faces resembling frequently encountered faces evoke not only a heightened sense of familiarity, but more favorable evaluations as well (Vokey and Read, 1992). Even more interestingly, own-ethnicity faces are known to receive higher ratings in terms of these items than other-ethnicity faces (Meissner et al., 2005). One way to interpret this is that familiarity with specific kinds of faces breeds more positive affects, which could also explain why all virtual faces appear more strange and unpleasant—or even eerie—than real human faces (Kätsyri et al., 2015). Another line of research has demonstrated that inversion can eliminate grotesqueness caused by distorted configural features. In particular, this seems to be the case for the so-called Thatcher illusion, in which eyes and mouth are flipped vertically (Stürzel and Spillmann, 2000). If typical human features are distorted in virtual faces, virtual faces should elicit less favorable evaluations than real human faces. Furthermore, if these features are at least partly configural in nature, inversion should reduce their effects. These two hypotheses are stated explicitly below.

H5: Virtual faces receive higher eeriness ratings than real faces.

H6: Inversion decreases the eeriness of virtual as compared with real faces.

### Methods

#### Participants

Participants were 64 (32 men and 32 women) university students or university graduates in the age range 18 to 36 years (*M* = 22.6 years). Participants were recruited via the SONA system of Maastricht University, flyers placed in the campus, and social media. Two original participants who scored high on PI20 prosopagnosia self-report questionnaire (Shah et al., 2015) and additionally received low overall scores in the present recognition memory task were excluded and replaced with new participants. Male and female participants did not differ statistically significantly on PI20 scores (*M* = 39.1 and 40.7, *SD* = 8.1 and 7.7), *T*_(62)_ = 0.84, *p* = 0.407. The majority (89%) of participants reported having played video games with realistic human-like characters at most once per month during the last year. That is, most participants had little experience with realistic virtual characters. All participants identified themselves as Caucasian in ethnic origin. Participants received a 7.5 € voucher in compensation for their participation. All participants gave written informed consent in accordance with the Declaration of Helsinki. The present studies were reviewed and approved by the ethics committee of the Faculty of Psychology and Neuroscience.

#### Stimuli

Research stimuli were 80 neutral face images (half female) from Glasgow (Burton et al., 2010) and Radboud (Langner et al., 2010) face image sets, replicated both as real and virtual versions. Face images were selected on the basis of distinctiveness preratings (cf. Valentine, 1991; McKone et al., 2007) from a larger set of 100 face images. These initial images were oval-masked and matched for luminance, contrast and colors but not for spatial frequency contents. Twenty-five participants who did not take part in the actual study rated the images for distinctiveness on a 7-step semantic differential scale ranging from “very typical/very difficult to recognize” to “very distinctive/very easy to recognize.” Twenty images were dropped on the basis of individual consideration and the remaining 80 images were divided evenly into eight stimulus sets based on their mean ratings. Finally, the selected images were replicated as real and virtual versions and matched for low-level features similarly as the strictly matched color images in Study 1 (Figure 1).

#### Procedure

The present study design was adapted from two previous OEB studies that included both face ethnicity and inversion as factors (Rhodes et al., 1989; Vizioli et al., 2011). In particular, participants completed standard recognition memory tasks separately for real and virtual faces, with the task order counterbalanced across participants. Recognition memory tasks for real and virtual faces were separated by a 2-min break. Both tasks consisted of a study and a test phase. During the study phase, participants were asked to view and memorize 20 faces presented in a pseudo-randomized order. Each face was presented for 5 s and preceded by a fixation cross for 2 s. All study faces were shown in upright orientation.

In the test phase, the 20 old faces (seen during the study phase) were interleaved with 20 new faces, and all faces were presented in a pseudo-randomized order. Half of the images were shown in upright orientation and the other half in inverted (rotated 180°) orientation. Participants were instructed to answer as quickly and as accurately as possible whether they had seen each face during the study phase or not using response buttons “S” and “L” on the keyboard. The assignment of response buttons was counterbalanced across participants. Each image remained on the screen until a response was received from the participant, and images were separated by 2-s fixation cross trials. Participants saw only real or virtual faces during the same study-test cycle. The eight stimulus sets were counterbalanced with the face type, trial type, and orientation conditions. Male and female participants were assigned evenly into counterbalancing conditions. Prior to the actual recognition memory tasks, participants practiced the study-test procedure with 20 faces which were not included in the actual study.

After the recognition memory tasks, participants were asked to evaluate how human-like and eerie the faces appeared on a 7-step Likert scale ranging from total disagreement to total agreement. Eeriness was defined as “being so mysterious, strange, or unexpected as to send a chill up the spine.” Participants rated the same 80 faces they had seen during the memory tasks, each with the same face type (real or virtual) and orientation (upright or inverted). To test whether the order of human-likeness and eeriness ratings would bias the results, participants gave these ratings in either separate blocks beginning from human-likeness (16 participants), in separate blocks beginning from eeriness (16 participants), or simultaneously in the same block (32 participants). In the former two conditions, human-likeness and eeriness were only explained prior to the beginning of their respective blocks. Male and female participants were assigned evenly into these conditions. All tasks were programmed and presented using E-Prime 2.0 (Psychology Software Tools, Pittsburgh, PA), and displayed on a 24” Asus VG248QE monitor.

#### Preprocessing

Hit and false alarm rates were transformed into *d*′ sensitivity and c response bias indices similarly as in Study 1. In this study sensitivity *d*′ reflects the extent to which participants were able to differentiate between old (seen during the study phase) and new (not seen) faces, whereas response bias *c* refers to the general tendency to respond “seen” or “not seen.” Positive c values refer to more conservative response criterion or a tendency to respond “not seen” for all faces, whereas negative values refer to more liberal response criterion or tendency toward responding “seen” for all faces.

### Results

#### Recognition of real and virtual faces

We used 2 × 2 within-subjects ANOVAs to analyze the effects of face type and orientation on sensitivity (*d*′) and response bias (*c*) indices on the one hand, and hit and false alarm rates on the other. We also tested whether any of these indices were influenced by the order of real and virtual face blocks but failed to observe any significant effects for block order or its interaction with face type (*p* > 0.270, $\eta_{p}^{2}$ < 0.02). This suggests that block order did not exert substantial generic or face type specific effects in the present study. Given that we had clear *a priori* predictions for our results, we did not adopt multiple-comparison correction in further analyses.

**Figure 3:**
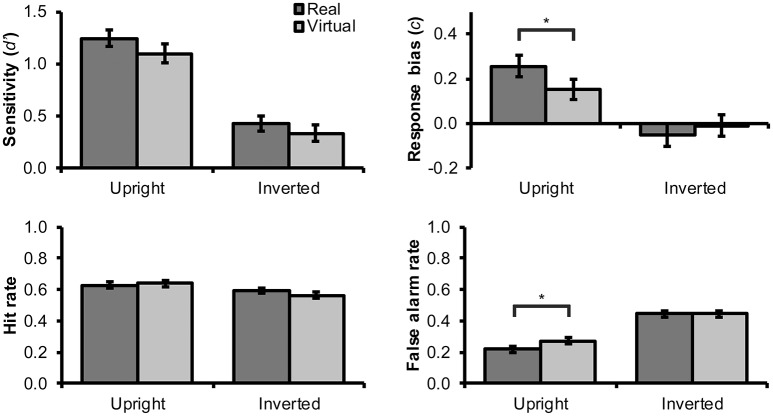
Mean (and SEM) values for *d*′ sensitivity index, *c* response bias index, hit rate, and false alarm rate for the recognition memory task. Statistically significant differences between virtual and human faces are denoted with an asterisk (“*”).

Recognition memory results are illustrated in Figure 3. Although visual inspection of this figure suggests that sensitivity scores were slightly higher for real as compared with virtual faces, as predicted by H1, this effect failed to reach statistical significance, *F*_(1, 63)_ = 2.75, *p* = 0.102, $\eta_{p}^{2}$ = 0.04.

Hypothesis H2 predicted a more lenient response bias (i.e., lower *c* scores) for virtual faces. Figure 3 suggests that response bias may have been less conservative for virtual than for real faces, but only in the upright condition. Given that face inversion exerted a considerable impairment on the processing of faces (see below), stronger response bias effects should in fact have been expected particularly for upright faces. In support, we observed a borderline significant interaction effect between face type and inversion, *F*_(1, 63)_ = 3.94, *p* = 0.052, $\eta_{p}^{2}$ = 0.059. Consequently, we decided to test H2 specifically for upright faces. This analysis confirmed a statistically significant and moderately large (Cohen, 1992) effect for face type in upright faces, *F*_(1, 63)_ = 4.40, *p* = 0.040, $\eta_{p}^{2}$ = 0.650, but not in inverted faces, *F*_(1, 63)_ = 0.78, *p* = 0.381, $\eta_{p}^{2}$ = 0.012.

Following the above logic, we next tested the effect of face type on false alarm rates in upright condition. In support of H3, our results demonstrated a significantly higher false alarm rate with a moderate effect size for virtual rather than real faces in upright orientation (see Figure 3), *F*_(1, 63)_ = 6.14, *p* = 0.016, $\eta_{p}^{2}$ = 0.089, but not in inverted orientation, *F*_(1, 63)_ = 0.00, *p* = 1.000, $\eta_{p}^{2}$ = 0.00. For hit rate, the effect of face type was not significant in either upright orientation, *F*_(1, 63)_ = 0.33, *p* = 0.568, $\eta_{p}^{2}$ = 0.005, or in inverted orientation, *F*_(1, 63)_ = 1.75, *p* = 0.191, $\eta_{p}^{2}$ = 0.027. These findings suggest that the more lenient response bias for upright virtual faces was driven mainly by false alarm responses, that is, participants' higher tendency to answer “seen before” to novel virtual faces. The 95% CI for the false alarm rate difference between virtual and real faces was [0.01, 0.10].

Inversion had a statistically significant and large effect on sensitivity, *F*_(1, 63)_ = 94.73, *p* < 0.001, $\eta_{p}^{2}$ = 0.601, and response bias, *F*_(1, 63)_ = 15.39, *p* < 0.001, $\eta_{p}^{2}$ = 0.196. As can be seen in Figure 3, inverted faces received lower sensitivity scores and more liberal response criterion (lower *c* scores). Looking at this the other way, inverted faces received moderately lower hit rates, *F*_(1, 63)_ = 5.58, *p* = 0.021, $\eta_{p}^{2}$ = 0.081, and much higher false alarm rates, *F*_(1, 63)_ = 78.67, *p* < 0.001, $\eta_{p}^{2}$ = 0.555, than upright faces (Figure 3, lower panels). For inverted and upright faces, the 95% CI for the false alarm rate difference was [0.15, 0.24].

Contrary to H4, the interaction effect between face type and inversion on *d*′ was not statistically significant, *F*_(1, 63)_ = 0.15, *p* = 0.700, $\eta_{p}^{2}$ = 0.002. Conversely, simple tests confirmed a significant and large inversion effect for both real, *F*_(1, 63)_ = 70.69, *p* < 0.001, $\eta_{p}^{2}$ = 0.529, and virtual faces, *F*_(1, 63)_ = 41.63, *p* < 0.001, $\eta_{p}^{2}$ = 0.398.

#### Self-report ratings

We first tested whether rating order (human-likeness first, eeriness first, or both together) had significant main or interaction effects for face type at a lenient significance threshold of *p* < 0.100. Because no significant effects were observed for either human-likeness (*p* > 0.128) or eeriness (*p* > 0.440), this confound variable was dropped from further analyses. Hence, self-report ratings were analyzed using a 2 (face type) × 2 (inversion) within-subjects ANOVA.

**Figure 4:**
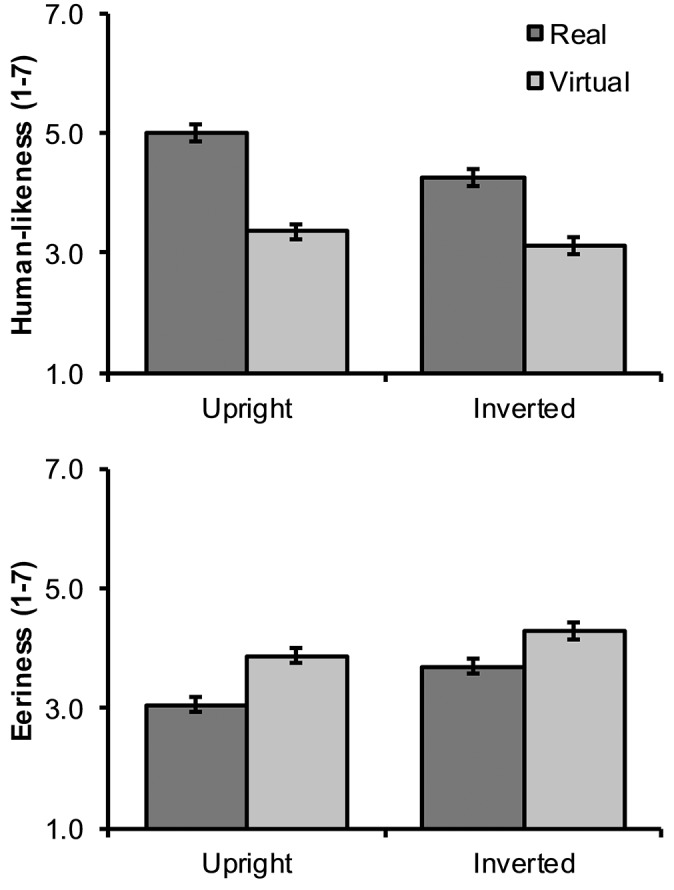
Mean (and SEM) human-likeness and eeriness ratings for upright and inverted, real and virtual faces.

Human-likeness and eeriness ratings are illustrated in Figure 4. Real as compared with virtual faces received significantly higher human-likeness ratings with a large effect size, *F*_(1, 63)_ = 78.41, *p* < 0.001, $\eta_{p}^{2}$ = 0.554. That is, similarly as in our pretest, participants were clearly able to discriminate virtual from real faces. There was also a significant interaction between face type and inversion such that inversion decreased the human-likeness difference between real and virtual faces (cf. Figure 4), *F*_(1, 63)_ = 31.25, *p* < 0.001, $\eta_{p}^{2}$ = 0.332. Looking at this the other way, real faces received lower human-likeness ratings when inverted (*p* < 0.001), whereas inversion did not have a statistically significant effect on virtual faces (*p* = 0.083).

In H5, we predicted that virtual faces would receive higher eeriness ratings than real faces. This prediction was confirmed, given that the difference between virtual and real faces was statistically significant and large, *F*_(1, 63)_ = 40.34, *p* < 0.001, $\eta_{p}^{2}$ = 0.390. Finally, in H6 we predicted that inversion would reduce or eliminate the eeriness of virtual faces. At first sight, this hypothesis appeared to receive support, given that the interaction between face type and inversion was significant with a moderate effect size, *F*_(1, 63)_ = 5.50, *p* = 0.022, $\eta_{p}^{2}$ = 0.080. However, as can be seen in Figure 4, inversion in fact increased rather than decreased eeriness for both virtual (*p* = 0.009) and real faces (*p* < 0.001). Apparently, the interaction effect was significant because this increase was greater for real rather than virtual faces and not because inversion decreased the eeriness of virtual faces in particular.

## General discussion

In the present investigation, we set to find out whether highly-realistic virtual faces tap perceptual expertise similarly as real human faces. Unlike faces of different ethnic groups in humans, virtual, and real faces tend to differ with respect to low-level visual features, which might contribute to differences in perceptual processing. In Study 1, we demonstrated that virtual faces can still be differentiated from real faces even after these two types of faces have been matched for spatial frequency contents, brightness, contrast, and colors. We interpret this to mean that individuals are able to use local features or their configurations to decipher whether a face is real or virtual. In Study 2, we showed that in a recognition memory task, virtual as compared with real faces elicit a less conservative response bias and a higher proportion of false alarms. Virtual and real faces did not differ with respect to discrimination accuracy or the magnitude of inversion effect, however.

The present findings resemble OEB findings in recognition memory studies with real human faces. Such studies have, however, typically identified a mirror pattern in which other-ethnicity faces receive both a lower proportion of hits and a higher proportion of false alarms than own-ethnicity faces (Meissner and Brigham, 2001). This mirror pattern has also been seen as lower discrimination sensitivity in the aggregate index that pits hits against false alarms. In contrast, we observed a difference in response bias but not in discrimination sensitivity. Given that the aggregate response bias measure depends positively on both hits and false alarms, and virtual as compared with human faces elicited a higher proportion of false alarms with a slight tendency toward higher proportion of hits as well (cf. Figure 3), this pattern of results is not surprising.

Importantly, the higher proportion of false alarms for virtual faces was predicted on the basis of the highly influential face-space model (Valentine, 1991; Valentine et al., 2016). Here the reasoning was that individuals' hypothetical face-space representation is optimized for real human faces, and that this representation is not necessarily appropriate for encoding virtual faces whose features or feature configurations differ from those of real faces. Similarly as for other- vs. own-ethnicity faces, differences between virtual faces are hence encoded imperfectly, which leads to a denser representation in the face-space. When individuals are making judgments in a recognition memory task, virtual faces then allegedly activate more face exemplars than equivalent real faces, which leads to a false sense of familiarity and a higher proportion of false alarms. The present study hence suggests that, similarly as other- vs. own-ethnicity faces, virtual faces tap perceptual expertise less efficiently than real faces. This effect is particularly evident in false alarm choices. The present study hence makes a contribution to existing research literature by demonstrating this theoretically predicted false alarm effect for virtual faces.

The present investigation is similar to that of Balas and Pacella (2015), given that both they (in their Experiment 1) and we (in Study 2) carried out a recognition memory task for real and virtual faces. The major difference between these studies is that we used stimuli that were matched for low-level features, spatial frequency contents in particular. The present results suggest that such matching eliminates the discrimination advantage for real faces observed by Balas and Pacella. In contrast, their results did not support different response bias or false alarm effects for real and virtual faces. We suggest that this difference originated from other methodological differences. First of all, the present study may have had higher statistical power for detecting a response bias effect because of a higher number of participants (64 against 18) and a within- rather than between-subjects design. Second, the response bias effect may have been more pronounced in the present study because of the less demanding recognition memory task (with 40 instead of 90 faces). The present investigation also differed from the study by Balas and Pacella because we studied inversion effects in a recognition memory task and considered the subjective evaluations of virtual and real faces.

Previous research evidence gives reason to believe that inversion effect is a hallmark of perceptual expertise for faces and other well-learned stimuli (Maurer et al., 2002), and that this effect is stronger for own- as compared with other-ethnicity faces (e.g., Rhodes et al., 1989). Unexpectedly, Balas and Pacella (2015; Experiment 2) demonstrated a similar inversion effect for real and virtual faces in a perceptual discrimination task, possibly due to ceiling effects in their results. The present study replicates this finding in a more difficult and different (recognition memory) task. If inversion effect is a hallmark of perceptual expertise, why did inversion then elicit roughly equal degradation on real and virtual faces? Similarly as Balas and Pacella (2015), we suggest that the human visual system processes virtual faces in a highly face-like manner. This statement is perhaps particularly uncontroversial for such highly realistic virtual stimuli as those used in the present study (cf. Figure 1). Inversion had a drastic overall effect on the proportion of false alarms (lower 95% CL for the difference 15 percentage units), which was clearly larger than the effect of face type in upright faces (upper 95% CL for the difference 10 percentage units). Hence, we suggest that inversion compromised face processing to the extent of concealing the more subtle processing differences between real and virtual faces.

Given that face inversion is thought to influence configural processing more than featural processing, the observed findings do not support the suggestion that virtual faces would be processed in a less configural manner than real faces. However, although this was not a specific aim in the present study, the human-likeness ratings from Study 2 suggest that configural and featural information may have played a different role on the recognition of human-likeness in the case of real and virtual faces. Specifically, our results showed that inversion elicited decreased human-likeness ratings for real faces but had lesser or no influence on virtual faces. This suggests that configural processing, which was impaired by inversion, was important for identifying real faces as human. On the other hand, virtual faces were still recognizable as non-human after inversion, plausibly because this judgement was mainly based on individual features. There is some previous evidence suggesting that eyes could be a particularly important feature for differentiating real from virtual faces (Looser and Wheatley, 2010).

Overall, the present self-report findings from Study 2 confirm the previous observation that virtual faces are always considered more eerie than real faces. The results also demonstrated that this difference is smaller for inverted than for upright faces. At first sight, this seemed to support the prediction that inversion can eliminate the eeriness of virtual faces similarly as with “Thatcherized” faces (Stürzel and Spillmann, 2000). However, a closer inspection of our results showed that inversion elicited increased eeriness for both real and virtual faces but that this increase was larger for real faces. It is plausible that the overall heightened eeriness for inverted faces reflected more effortful processing caused by increased encoding error (Valentine et al., 2016). Furthermore, human-likeness ratings suggested that inversion had a larger effect on the categorization of real as compared with virtual faces. Given that eeriness ratings closely parallel these findings, it is possible that inversion had a differential effect on real and virtual faces simply because inverted real faces were more difficult to recognize as human than upright real faces. Hence, the present findings cannot be taken as support for the prediction that inversion would eliminate the eeriness of virtual faces by reducing configural differences between virtual and real faces.

We want to address some potential limitations of the present investigation and to suggest directions for future research. First, similarly as Balas and Pacella (2015) and Crookes et al. (2015), we used FaceGen software as the basis for our virtual stimuli. However, unlike them, we additionally matched virtual and real faces with respect to various low-level visual features. It could be argued that after this matching, the present virtual stimuli were no longer representative of typical virtual faces. We want to emphasize, however, that the above two studies have already demonstrated the limits of typically used stimuli (e.g., those generated by FaceGen), and that our aim was instead to test whether real and virtual faces are still processed differently after they have been matched for most obvious low-level visual confounds. Hence, the important question is not whether our stimuli were high in mundane realism (i.e., whether they were similar to modern computer-rendered faces) but whether they were high in psychological realism (i.e., whether they tapped psychological processes relevant for perceiving animacy in faces) (cf. Shadish et al., 2002). This question was addressed in Study 1, which clearly showed that the present stimuli were perceived distinctly as human and non-human stimuli.

Nevertheless, we want to acknowledge other confounds that could still have influenced the present stimuli even after the matching procedure. Because virtual stimuli were generated by replicating real faces in the FaceGen software's parametric space, it is possible that virtual faces or some of their features (e.g., nose shapes) might have been more similar to each other than was the case for original faces. This reduced variability could then trivially explain the inflated false alarm rate for virtual faces. We also note that featural matching was only done at the global level, that is, across whole images. After such global matching, local features might still have had for example varying brightness levels (for example, darker nose region in one image and darker skin region in the other). With more detailed local-level matching, however, maintaining whole-image consistency would have become a practical impossibility. Given these shortcomings, we cannot fully exclude the possibility that the present results were still specific to the present stimuli. We suggest that this problem in fact applies to all studies using virtual stimuli, given the obvious impossibility of creating virtual faces that are visually identical to real faces yet at the same time discriminable from them. Future studies might want to consider using more than one method for producing virtual stimuli to increase the generalizability of their results; however, even this approach does little to solve the fundamental problem related to the lack of unequivocal operationalization of “virtual” or “artificial” stimuli.

An ideal solution to this problem might be to keep the stimuli constant but to present them in varying contexts. We give some suggestions for future research, which at the same time refine the present research questions. First, the effect of perceptual expertise could be tested directly by training participants with either virtual or real faces before the experimental task, for example by adopting a similar training paradigm as Burleigh and Schoenherr (2015). Second, perceptual expertise could also be tested by preselecting participants with high or low exposure to realistic virtual faces in video games and other digital media. Third, future studies could test whether the processing of virtual faces is prone to similar social-cognitive and motivational factors as other-ethnicity and out-group faces (see Young et al., 2012). For example, Bernstein et al. (2007) demonstrated that merely assigning other people as in-group vs. out-group members—for example, members of the same or other universities—elicits higher discrimination sensitivity in a recognition memory task. Similarly, labeling the same ambiguous real/virtual faces (cf. Cheetham et al., 2011) or even the same human faces as either real or virtual might provoke different processing strategies in individuals. Importantly, all of these hypotheses can be tested by holding the same stimuli constant, which eliminates the influence of visual differences on obtained results.

We would also like to note that performing recognition memory task separately for real and virtual faces could possibly have elicited different processing strategies, which could then have inflated existing response bias differences between them. This effect would in fact resemble the effect of arbitrary labeling as hypothesized above, and it would mean that the present response bias finding was related more to social-cognitive processes than to visual differences between the stimuli. Future studies are required to explore this possibility, however. In particular, the present study could be replicated by interleaving virtual and real faces within the same blocks.

To summarize, the present findings show that virtual faces evoke a higher proportion of false alarms than real faces in a recognition memory task, which suggests that virtual faces do not tap face processing expertise to the same extent than real faces. Furthermore, the present findings suggest that this literal lack of familiarity might then contribute to the uneasiness or even eeriness virtual faces trigger in human observers, which was also observed in the present investigation. The present investigation makes a significant contribution to previous literature by considering low-level visual confounds in the stimuli, by demonstrating that the differential processing of virtual and real faces is particularly evident in false alarm choices, and by linking this result to the qualitative evaluation of virtual faces.

## Author contributions

JK designed and implemented the experiment, analyzed the data, and wrote the manuscript.

### Conflict of interest statement

The author declares that the research was conducted in the absence of any commercial or financial relationships that could be construed as a potential conflict of interest.
